# Jejunal Adenomatous Polyp Presenting as Intussusception in Peutz-Jeghers Syndrome With von Hippel-Lindau (VHL) Mutation Carrier Status

**DOI:** 10.7759/cureus.111165

**Published:** 2026-06-19

**Authors:** Kyra Salinkas, Sabeen Wazir, Madeline Keen, Lance Albin

**Affiliations:** 1 Medicine, Edward Via College of Osteopathic Medicine, Monroe, USA; 2 General Surgery, Saint Francis Medical Center, Monroe, USA

**Keywords:** adenomatous polyps, hamartomaous polyp, intussusception, peutz-jegher’s syndrome, small-bowel obstruction, von hippel-lindau disease

## Abstract

Peutz-Jeghers Syndrome (PJS) is a rare inherited autosomal dominant condition characterized by hamartomatous polyps in the gastrointestinal (GI) tract and mucocutaneous hyperpigmentation involving the lips, oral mucosa, and hands. Patients with PJS are at an increased risk for intussusception, causing small bowel obstruction (SBO), a surgical emergency. A 29-year-old female presented to the emergency department (ED) with stabbing left lower back pain that began the previous night and progressively worsened, accompanied by nausea. Her medical history was significant for PJS, von Hippel-Lindau (VHL) mutation carrier status, prior SBO requiring surgical resection at 14 years of age, recurrent left lower quadrant (LLQ) intussusception, and rectal bleeding. On physical examination, the patient had LLQ abdominal tenderness and left costovertebral angle tenderness. CT imaging of the abdomen revealed findings concerning for residual or recurrent LLQ intussusception. An exploratory laparotomy was recommended with resection of approximately 20 cm of the jejunum, followed by reconstruction with a stapled side-to-side anti-peristaltic anastomosis. Pathology demonstrated a tubular adenomatous polyp associated with intussusception and ischemic injury with no evidence of malignancy or high-grade dysplasia. This case highlights a unique presentation of a jejunal adenomatous polyp causing intussusception in a patient with PJS. Awareness of this atypical polyp location in a patient with PJS is essential, given its potential for malignancy and implications for management and surveillance. Furthermore, the patient’s known status as a VHL mutation carrier emphasizes the importance of comprehensive assessment in patients with overlapping tumor syndromes.

## Introduction

Peutz-Jeghers syndrome (PJS) is a rare inherited autosomal dominant polyposis syndrome characterized by hamartomatous polyps in the gastrointestinal (GI) tract and mucocutaneous hyperpigmentation involving the lips, oral mucosa, and hands [1,2]. It is most often caused by a germline mutation in the serine/threonine kinase 11 (STK11/LKB1) tumor suppressor gene located on chromosome 19 [1]. Loss of STK11 function causes abnormal cell proliferation, leading to the formation of hamartomatous polyps [1]. Histologically, these polyps demonstrate a characteristic branching, arborizing core of smooth muscle derived from the muscularis mucosae, lined by normal epithelium [2]. Larger polyps contain more smooth muscle fibers, whereas smaller polyps exhibit relative epithelial overgrowth with branching crypts and minimal inflammation. This contrasts with juvenile polyps, another hamartomatous polyposis syndrome, characterized by abundant stroma between dilated cystic crypts with several inflammatory cells [3]. PJS polyps are most frequently found in the jejunum and ileum but can occur in the stomach and large intestine [1].

Polyp-related complications include rectal bleeding leading to anemia, intussusception, and bowel obstruction [1]. In contrast to pediatric cases, adult intussusception often has a definable pathologic lead point, usually requiring surgical intervention with removal of the telescoped bowel segment. If left untreated, it can lead to bowel obstruction, perforation, ischemia with subsequent necrosis, and sepsis [4]. Surveillance of the small intestine in adults with a polyposis syndrome is recommended every two to three years to lower the risk of intussusception and associated complications. Furthermore, patients with PJS are at an increased risk for a variety of malignancies, including colorectal, gastric, pancreatic, gynecologic, and breast cancers [5]. The estimated lifetime risk for developing any cancer exceeds 90% with a relative risk of approximately 15 compared to the general population [5,6].

Hamartomatous polyps are the hallmark of PJS. However, patients may develop other polyp types throughout the GI tract. Polyps with adenomatous changes, which carry higher malignancy potential, occur more frequently in the colon [7]. We report a unique case of a patient with a history of PJS and von Hippel-Lindau (VHL) mutation carrier status with recurrent intussusception secondary to a jejunal adenomatous polyp, managed with jejunal resection via open laparotomy.

This article was previously presented as a meeting poster at the Edward Via College of Osteopathic Medicine Research Day on February 27, 2026. 

## Case presentation

A 29-year-old Caucasian female presented to the emergency department (ED) with sudden, intermittent stabbing left lower back pain. She reported that it began the previous night and progressively worsened with nausea. She denied vomiting. Upon chart review, the patient has a history of admission for this complaint and has been admitted three times in the last four months. The most recent admission, one month prior, the patient underwent a double balloon enteroscopy, leading to the removal of four polyps. This was complicated by rectal bleeding and anemia requiring hospitalization and a transfusion of three units of packed red blood cells. Computed tomography (CT) imaging of the patient’s abdomen at the time revealed intussusception that subsequently resolved with conservative management. Additionally, it was noted that the left lower quadrant (LLQ) had a large enhancing solid mass. Her medical history was significant for PJS, VHL mutation carrier status, prior small bowel obstruction (SBO) requiring surgical resection at 14 years of age, recurrent LLQ intussusception, end-stage renal disease (ESRD) requiring hemodialysis with anemia and secondary hyperparathyroidism, hypertension, and anxiety.

On physical examination, the patient had LLQ abdominal tenderness and left costovertebral angle tenderness. She denied any blood in her stool. Initial laboratory studies revealed anemia and elevated renal markers indicating ESRD, thus the patient was scheduled for same-day dialysis (Table 1). An X-ray of her lumbar spine and abdomen was performed, but no acute findings were noted. CT imaging of the abdomen without contrast revealed findings concerning for residual or recurrent LLQ intussusception of the small bowel in the lower left abdomen or upper pelvis (Figure 1). This was separate from an area of suspected dystrophic sutural calcification likely from her previous surgical resection (Figure 2). Other findings included decreased renal size bilaterally and cortical and partial medullary calcifications bilaterally. 

**Table 1:** Initial Laboratory Report From Presentation in the Emergency Room. Reference ranges are based on standard adult laboratory values.

Laboratory Parameter	Patient Value	Reference Range	Interpretation
Hemoglobin	9.1 g/dL	12.0-16.0 g/dL	Decreased
Hematocrit	29.9%	36-46%	Decreased
Creatinine	9.11 mg/dL	0.6-1.2 mg/dL	Increased
Blood Urea Nitrogen (BUN)	42 mg/dL	7-20 mg/dL	Increased
Urine Pregnancy Test	Negative	Negative	Normal

**Figure 1:**
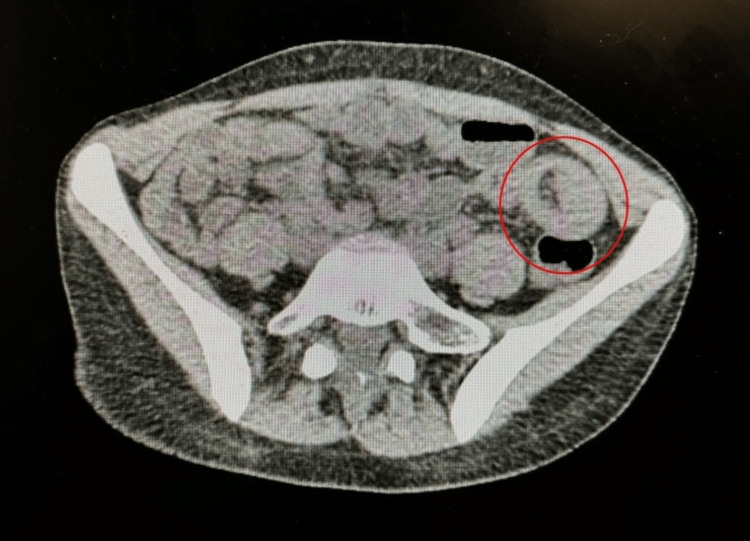
CT without contrast of abdomen indicating suspected area of intussusception. Red circle indicates intussusception.

**Figure 2:**
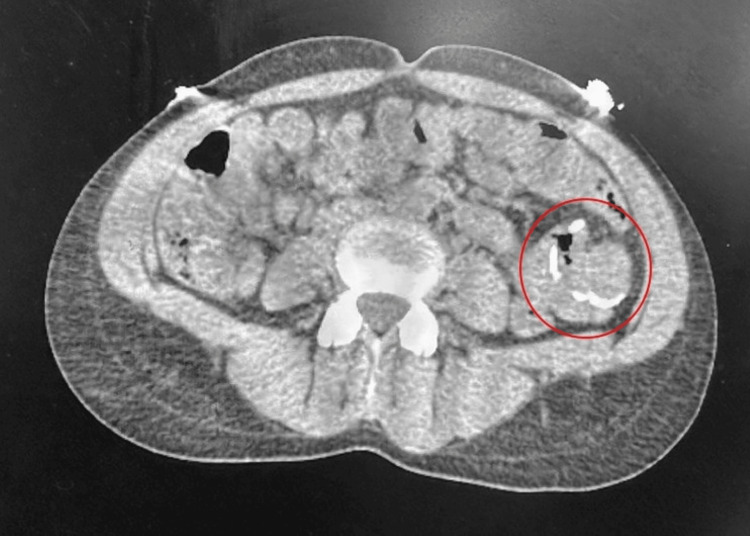
CT without contrast of abdomen displaying dystrophic sutural calcification from previous surgical resection. Red circle indicates dystrophic sutural calcification.

After consideration of the patient’s symptoms, past medical history, imaging, and previous hospitalizations, general surgery was consulted. An exploratory laparotomy was recommended with resection of the affected small intestine.

The open laparotomy was carefully performed using her previous midline incision site to minimize damage to surrounding healthy tissue and the development of additional adhesions. The fascia was opened with blunt finger dissection, and the ligament of Treitz was identified. The small intestine was inspected, and the section of intussusception was located in the middle of the jejunum. A mass was noted within the intussuscepted bowel. Windows in the mesentery were made proximally and distally, then the intestine was divided with a 60 mm linear stapler with green reload. The mesentery was removed with Enseal (Ethicon, Raritan, NJ, USA). Total resection of the involved jejunum measured approximately 20 cm. The specimen was sent to pathology. Reconstruction of the bowel was completed with a stapled side-to-side anti-peristaltic anastomosis with a 60 mm linear stapler with green reload. The common enterotomy was closed with 3-0 V-Lok suture (Medtronic, Minneapolis, MN, USA) in two layers. The mesenteric defect was closed with 3-0 Vicryl. The skin was closed with 4-0 Monocryl in a running fashion and Dermabond (all three products from Ethicon). The patient was returned to anesthesia, extubated, and taken to the post-anesthesia care unit for recovery.

The patient recovered well postoperatively. She was started on a clear liquid diet and advanced as tolerated. At her two-week post-operative follow-up, she reported improvement in her abdominal discomfort with regular bowel movements. Pathology demonstrated a tubular adenomatous polyp associated with intussusception and ischemic injury with no evidence of malignancy or high-grade dysplasia.

## Discussion

PJS is an autosomal dominant polyposis syndrome where the incidence can range from one in 50,000 to one in 200,000 live births. In patients with PJS, one study found that approximately 88% had typical PJS hamartomatous polyps [8]. Other polyp types can arise in patients with PJS, including gastric hyperplastic-like, dysplastic, or, as seen in our patient, adenomatous polyps. However, adenomatous polyps in PJS are typically seen in the colon rather than the jejunum. Therefore, when PJS-associated adenomatous polyps occur in the colon, they can create confusion with the genetic condition familial adenomatous polyposis [7].

Although the malignant potential for hamartomatous polyps remains uncertain, adenomatous polyps are recognized for their high risk of malignant progression [5,9]. Regardless, continued surveillance remains the mainstay of syndrome management. Patients and at-risk family members are recommended to start screening at an early age with a colonoscopy, small bowel examination, and esophagogastroduodenoscopy beginning at the age of eight, with follow-up every two to three years. Gynecologic surveillance, including pelvic examination and Pap smear, is recommended beginning at the age of 18 and repeated annually. Mammography is recommended beginning at the age of 30 and repeated annually, along with biannual self-examinations. In males, testicular examination and ultrasonography are recommended beginning at the age of 10 and repeated annually [9].

In a PJS patient who also carries a VHL mutation, surveillance is imperative. VHL is another rare, primarily autosomal dominant disease caused by a mutation in the VHL gene on chromosome 3. It is characterized by hemangioblastomas of the central nervous system, clear cell renal carcinomas or cysts, pheochromocytomas, pancreatic cysts, neuroendocrine tumors, and endolymphatic sac tumors [10,11]. VHL is a tumor suppressor gene that follows Knudson’s two-hit hypothesis, causing disease onset. In addition to the patient’s PJS surveillance, our patient needs to follow up with recommended screenings for VHL. These include ophthalmologic examinations, abdominal ultrasonography, audiology assessments, magnetic resonance imaging (MRI) of the brain and spine, and testing for pheochromocytoma [11]. In one study, it was found that overall penetrance was 87% at the age of 60 [12].

This case highlights the complexity of managing a patient with overlapping hereditary tumor syndromes. The presence of a tubular adenomatous polyp in our patient, causing recurrent intussusception, emphasizes the importance of routine surveillance with management of any findings and strong multidisciplinary collaboration. Awareness of this atypical polyp with high malignant potential and its unusual location is essential in guiding future surveillance strategies.

## Conclusions

This case highlights a unique presentation of a jejunal adenomatous polyp causing intussusception in a patient with PJS and VHL mutation. It calls attention to the importance of prompt surgical intervention and careful histologic evaluation of polyps. Comprehensive assessments in patients with overlapping tumor syndromes require multidisciplinary care and timely interventions to reduce morbidity and improve patient outcomes. Future research should focus on optimizing small intestine surveillance strategies. Improvements could allow catching more of these missed atypical polyps in PJS patients, but also any polyps in those with polyposis syndromes.
